# Using shock generator for the fuel mixing of the extruded single 4-lobe nozzle at supersonic combustion chamber

**DOI:** 10.1038/s41598-024-57103-0

**Published:** 2024-03-17

**Authors:** As’ad Alizadeh, Dheyaa J. Jasim, Neaman Sohrabi, Mohsen Ahmed, S. Abdul Ameer, Safaa Mohammed Ibrahim, Hasan Khalid Dabis, Ali Adhab Hussein, Abbas J. Sultan

**Affiliations:** 1 Department of Civil Engineering, College of Engineering, Cihan University-Erbil, Erbil, Iraq; 2 Department of Mechanical Engineering, College of Engineering, Urmia University, Urmia, Iran; 3 Department of Petroleum Engineering, Al-Amarah University College, Maysan, Iraq; 4 Department of Mechanical and Mechatronics Engineering, Southern Illinois University, Edwardsville, IL 62026 USA; 5 Imam Abdulrahman Bin Faisal University, P.O. Box 1982, Dammam, 31441 Eastern Province Kingdom of Saudi Arabia; 6 Department of Automobile Engineering, College of Engineering, Al-Musayab University of Babylon, Hillah, Iraq; 7 Department of Optical Techniques, Al-Noor University College, Mosul, Iraq; 8 Ahl Al Bayt University, Kerbala, Iraq; 9 Department of Medical Laboratory Technics, Al-Zahrawi University College, Karbala, Iraq; 10 Department of Chemical Engineering, University of Technology-Iraq, Baghdad, Iraq; 11 Department of Chemical and Biochemical Engineering, Missouri University of Science and Technology, Rolla, MO 65409-1230 USA

**Keywords:** CFD, Combustion chamber, Fuel mixing, Extruded nozzle, Aerospace engineering, Mechanical engineering

## Abstract

The importance of the fuel injection configuration on the propulsion efficiency of high-speed vehicles is apparent. In this article, the use of an annular extruded 4-lobe nozzle for the injection of fuel jet in a supersonic combustor of a scramjet engine in the existence of a shock generator is examined. The main aim of this study is to obtain the efficient jet arrangement for efficient fuel mixing inside the engine of hypersonic vehicles. A numerical approach is used to model the supersonic air stream and cross-jet flow with the SST turbulence model. The role of nozzle altitude and internal air jet on the fuel mixing of the hydrogen within the high-speed domain are disclosed. The importance of the horseshoe vortex and counter-rotating vortex on the fuel distribution is also presented. Our results show that the usage of a coaxial jet instead of an annular jet would increase fuel mixing by more than 40% in the combustion chamber.

## Introduction

To improve fuel injection in a combustion chamber and enhance vortex generation, a shock generator can be employed. The shock generator is a device that utilizes the principle of shock waves to enhance the atomization and mixing of fuel with air. It introduces controlled shock waves into the fuel flow, leading to improved fuel breakup and more efficient combustion. Here is a general overview of the mechanism of a shock generator concerning fuel injection and vortex generation in a combustion chamber^1,2^.

The fuel injection process involves introducing fuel into the combustion chamber in a controlled manner^3,4^. Traditionally, a single transverse jet configuration is utilized, where fuel is injected perpendicular to the main airflow. The shock generator is integrated into the fuel injection system to enhance the atomization and dispersion of the fuel^5,6^.

The shock generator consists of a specially designed nozzle or a set of nozzles that create controlled shock waves in the fuel flow. These shock waves are generated by abrupt changes in the flow area or by using obstacles or diaphragms within the nozzle^7,8^. The shock waves propagate through the fuel, leading to rapid pressure and velocity changes.

The shock waves generated by the shock generator cause significant turbulence and disturbance in the fuel flow. This turbulence results in the breakup of larger fuel droplets into smaller droplets, enhancing the atomization process. Smaller fuel droplets have a larger surface area, promoting better mixing with air and improving combustion efficiency^9–11^.

The shock-induced turbulence and atomization contribute to the generation of vortices within the combustion chamber. Vortices are swirling motions of the fuel–air mixture that help in creating a more homogeneous mixture and improving the combustion process. The vortices increase the mixing of fuel and air, enhancing the combustion efficiency, and reducing emissions^12,13^.

The improved atomization and vortex generation facilitated by the shock generator result in better fuel–air mixing and a more uniform distribution of the mixture within the combustion chamber. This leads to more efficient and complete combustion, promoting better fuel efficiency and reduced emissions^14–16^.

By utilizing a shock generator in the fuel injection system, the atomization of the fuel is enhanced, and vortices are generated, leading to improved combustion characteristics^17–19^. The specific design and implementation of the shock generator may vary depending on the engine and application. Computational fluid dynamics (CFD) simulations and experimental testing are often employed to optimize the shock generator's configuration and parameters for maximum fuel injection improvement and vortex generation in a particular combustion chamber setup^20–22^.

In the context of fuel mixing behind a cross jet**,** different vortex types, including the horseshoe vortex, play a significant role in influencing the mixing process. The horseshoe vortex is a specific type of vortex that forms near the edges of a solid body, such as a fuel injector, in the presence of a cross jet^23,24^. Here's a closer look at the role of different vortex types, including the horseshoe vortex, on fuel mixing behind a cross jet:

The horseshoe vortex forms near the leading edge of a solid body, such as a fuel injector, when a cross jet interacts with it. It consists of a pair of counter-rotating vortices that resemble the shape of a horseshoe^25,26^. The horseshoe vortex plays a crucial role in enhancing fuel mixing by inducing significant fluid motions and promoting interaction between the cross jet and the fuel plume^27^.

In the wake of the fuel injector, vortex shedding occurs. This phenomenon involves the formation and shedding of vortices downstream of the fuel injection point. These vortices can enhance fuel mixing by convecting the fuel and promoting its dispersion into the surrounding air^28,29^. The periodic shedding of vortices can result in turbulent mixing, leading to improved fuel–air homogenization.

Vortex roll-up is another important mechanism that contributes to fuel mixing behind a cross jet. As the cross jet interacts with the surrounding air, it induces the formation of roll-up vortices. These vortices develop due to the shear between the high-velocity cross jet and the surrounding air. Vortex roll-up enhances fuel–air mixing by entraining and stretching the fuel plume, facilitating better fuel dispersion and interaction with the cross-flowing air^30,31^.

Coherent structures refer to organized patterns of fluid motion within the fuel–air mixing zone. These structures, which can include vortices and other flow patterns, are responsible for enhancing the mixing process^32,33^. They help in transporting the fuel into regions of the flow with varying velocities, promoting better fuel dispersion and homogenization.

By promoting fluid motion, inducing vortex shedding, and facilitating vortex roll-up, different vortex types, including the horseshoe vortex, contribute to enhanced fuel mixing behind a cross jet^34,35^. These mechanisms increase the interaction between the fuel and the cross-flowing air, leading to improved fuel dispersion, better fuel–air homogenization, and ultimately, more efficient combustion. Understanding and optimizing the effects of various vortex types is crucial for optimizing fuel injection strategies and achieving optimal combustion performance in practical applications^36,37^.

The type of nozzle can have an impact on vortex generation. Different nozzle designs can influence the flow characteristics and the formation of vortices. Nozzles with specific geometries, such as contoured or convergent-divergent (CD) nozzles, are often used to enhance vortex generation. These nozzles are designed to optimize the flow expansion and accelerate the exhaust gases, which can lead to the formation of stronger vortices^38,39^. Nozzles with specific geometries, such as confined or convergent-divergent (CD) nozzles, are designed to enhance vortex generation for improved fuel mixing in supersonic combustors. These nozzles can create high-speed jets that interact with the surrounding flow, leading to the formation of strong vortices. The confined nozzle, for example, is characterized by a sudden expansion of the flow, which can induce a shock wave and create a recirculation zone that promotes mixing. On the other hand, the CD nozzle has a diverging section that can further stretch and mix the flow. The design of these nozzles is critical for optimizing the mixing process and ensuring efficient combustion in supersonic engines.

For example, CD nozzles feature a converging section followed by a diverging section. As the flow passes through the converging section, it accelerates, and when it reaches the throat, it reaches its maximum speed. As the flow expands in the diverging section, vortices can form due to the pressure gradients and flow separation. Additionally, the presence of chevrons or serrations on the nozzle walls can also affect vortex generation. These devices create disturbances in the flow, promoting the mixing of the exhaust gases and facilitating the formation of vortices^40–42^.

It's important to note that the specific effect of nozzle type on vortex generation can vary depending on the intended application and the specific flow conditions. Computational fluid dynamics (CFD) simulations and experimental studies are often employed to investigate and optimize nozzle designs for desired vortex characteristics. In a supersonic combustion chamber with a transverse fuel jet, the presence of a shock generator can induce various types of shocks that significantly influence the combustion process. These shocks play a crucial role in enhancing fuel–air mixing, promoting combustion efficiency, and stabilizing the flame^43–45^.

One common type of shock-induced in this configuration is the oblique shock wave. When the transverse fuel jet interacts with the incoming supersonic airflow, it can create an oblique shock wave. This shock wave compresses and slows down the airflow, generating a high-pressure region behind the shock. The oblique shock wave also causes the airflow to change direction, leading to the formation of a recirculation zone. This recirculation zone facilitates the mixing of the fuel and air, promoting combustion. Another important shock phenomenon is the bow shock wave. As the transverse fuel jet penetrates the supersonic airflow, it creates a bow shock wave in front of the jet. The bow shock wave compresses the incoming airflow, further enhancing the pressure and temperature in the region. This compression aids in fuel atomization and vaporization, improving fuel–air mixing and combustion efficiency. Additionally, the transverse fuel jet can induce normal shocks. These shocks occur when the fuel jet abruptly slows down the supersonic airflow, resulting in a sudden increase in pressure and temperature. The normal shocks contribute to the compression and heating of the fuel–air mixture, facilitating combustion^46^.

The presence of a shock generator, such as a strut or ramp, can enhance the generation and control of these shocks. These devices are strategically placed within the combustion chamber to manipulate the airflow and induce desired shock patterns. By adjusting the geometry and position of the shock generator, the strength and location of the shocks can be tailored to optimize fuel–air mixing and combustion efficiency. It is worth noting that the specific characteristics and interactions of shocks in a transverse fuel jet configuration with a shock generator depend on various factors, including the geometry of the chamber, fuel properties, and operating conditions. Computational simulations and experimental studies are often employed to analyze and optimize the shock-induced phenomena for improved combustion performance^47^.

A shock generator can promote efficient fuel–air mixing by creating oblique shocks, bow shocks, and normal shocks. These shocks compress and heat the airflow, leading to improved atomization and vaporization of the fuel. This enhanced mixing contributes to more complete combustion and increased combustion efficiency.

The presence of a shock generator can provide flame stabilization by creating recirculation zones and high-pressure regions. These regions help anchor and stabilize the flame, preventing flame blowout and ensuring sustained combustion.

**Figure 1:**
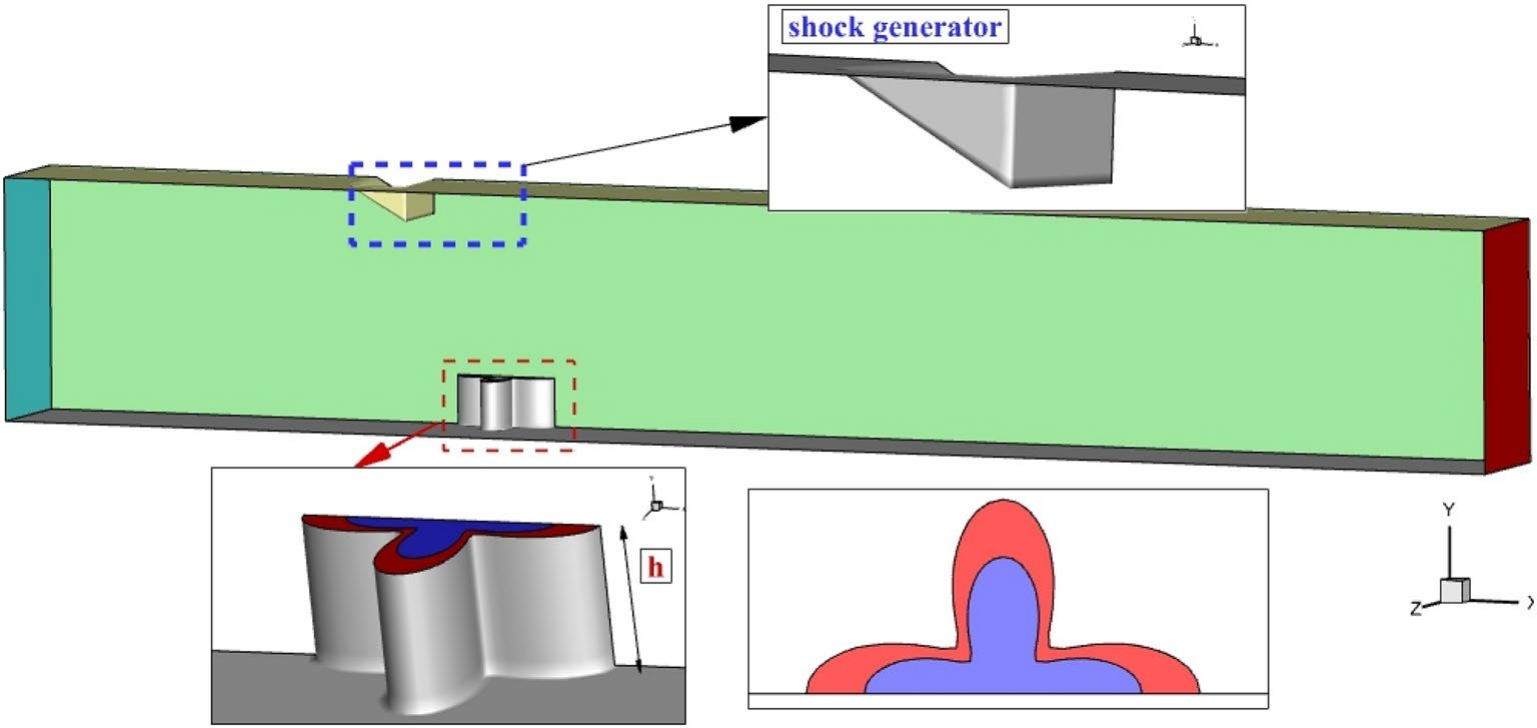
Proposed 4-lobe nozzle.

The main outline of this research is to investigate the usage of an extruded injector as a new jet configuration for fuel distribution inside the combustion chamber. In this method, the extruded nozzle increases the interaction by producing the upstream circulation which trails as a horseshoe vortex. In the proposed technique, the usage of the erected single 4-lobe nozzle in the existence of the shock produced by the upward nozzle is extensively analyzed via computational technique (Fig. 1). The influence of extruded nozzle tallness on the mechanism of the fuel interactions is widely examined. In addition, the annular nozzle is investigated along with the internal core of the air jet for evaluation of the role of higher interactions on the fuel mixing.

## Computational methodology

Numerical technique of computational fluid dynamic is used extensively in different applications^47–51^. The simulation of the cross-jet flow at the supersonic free stream is done by solving RANS equations with the SST turbulence model based on the previous works^44,46,52^. The assumption of the ideal gas is also done in the modeling of a transverse fuel injection system when fuel mixing is considered. In addition, the fuel jet is hydrogen gas and the free stream is supersonic flow. For the modeling of the secondary gas species, species transport is required. The energy equation is also coupled with the main mass and momentum equations by the reason of the produced shock wave of the shock producer. Since this study just focused on fuel distribution, reactions are not considered in the modeling of the proposed injection system.

Free stream air enters to domain from the left side with Mach = 4, Tinf = 1000 K, and Pinf 1 atm. Half of the proposed configuration is modeled since the domain is symmetry (green plane in Fig. 1). The shock generator is located at the top of the injector with an angle of 30°. A single annular extruded 4-lobe injector is selected in which the air jet is released from the inner nozzle as demonstrated in Fig. 1. Hydrogen gas with sonic velocity and 10% of the total pressure of free stream is released from the annular nozzle and the air jet with the same condition is injected from the internal nozzle. The area of the annular and internal nozzle is equal to the circular nozzle with a diameter of 2 mm. The length and width of annular nozzle is 4.4 mm and 2.6 mm. The length of the domain is 100 mm while the width and height of the domain are 4 mm and 10 mm. Two heights of 2 mm and 4 mm are examined in this article.

As depicted in Fig. 1, the annular jet nozzle is colored red while inner nozzle is defined by blue color. As described before, both nozzle have identical area. In the annular configuration, fuel jet is injected just from outer nozzle while the inner air jet and outer fuel jet are active in coaxial configuration.

**Figure 2:**
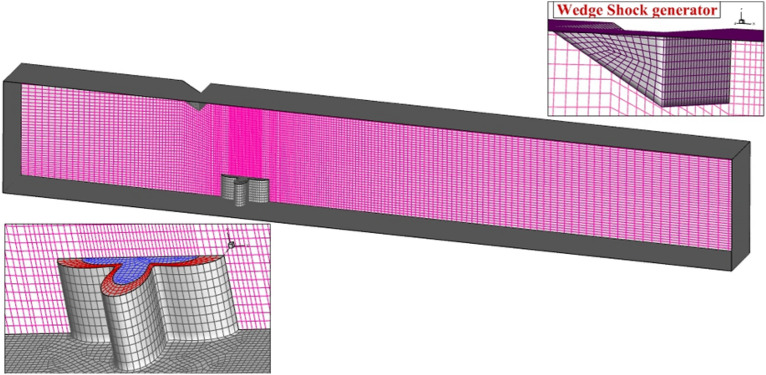
Grid production.

The grid generation for the extruded 4-lobe nozzle at supersonic flow with a shock producer at the top of the domain is displayed in Fig. 2. The size of the produced grid is adjusted based on the importance and gradient of the flow parameters in the proposed jet configurations. Since the expected shock waves happen near the injector and the wedge shock generator, grids with higher resolution are created in this section. As demonstrated in Fig. 2, the size of the grid near the outlet and inlet is lower than in other regions due to the uniform structure of the flow in these regions. In addition, the grid analysis for evaluation of the independence of the achieved results from the produced grid is done and its results are available in Table 1. The third grid is selected for the present investigation. To adopt grid based on turbulence model, y + is kept blow 8 in the first layer of generated grids.

**Table 1:** Grid study.

	Cells	Grid cells. along X, Y and Z direction	Hydrogen fraction (at 30 mm downstream)
Coarse	970,000	194 × 100 × 50	0.281
Medium	1,684,000	208 × 135 × 60	0.302
Fine	2,508,000	224 × 160 × 70	0.306
Very fine	4,320,000	270 × 200 × 80	0.307

## Results and discussion

The results of this study are also compared with the previous experimental and computational studies to authenticate the correctness of the applied methodology. For validation of the results, we selected similar jet configuration with experimental text to ensure the correctness of the results. The selected configuration is simple circular nozzle with diameter of 2 mm at supersonic cross flow. The comparison of the penetration height with experimental data^52^ and computational results is done in Table 2. The deviation of our results with experimental and computational data is less than 9.5% which is acceptable for the modeling of the flow in the combustor.

**Table 2:** Validation of penetration height.

Downstream (mm)	Numerical data of Pudsey et al. (mm)	Experimental data of Roger. (mm)	Present simulation (mm)
10	8.0	8.1	7.9
20	8.5	8.6	8.2
30	8.6	9.3	8.7
40	9.6	9.8	9.4
50	9.9	10.2	9.7

**Figure 3:**
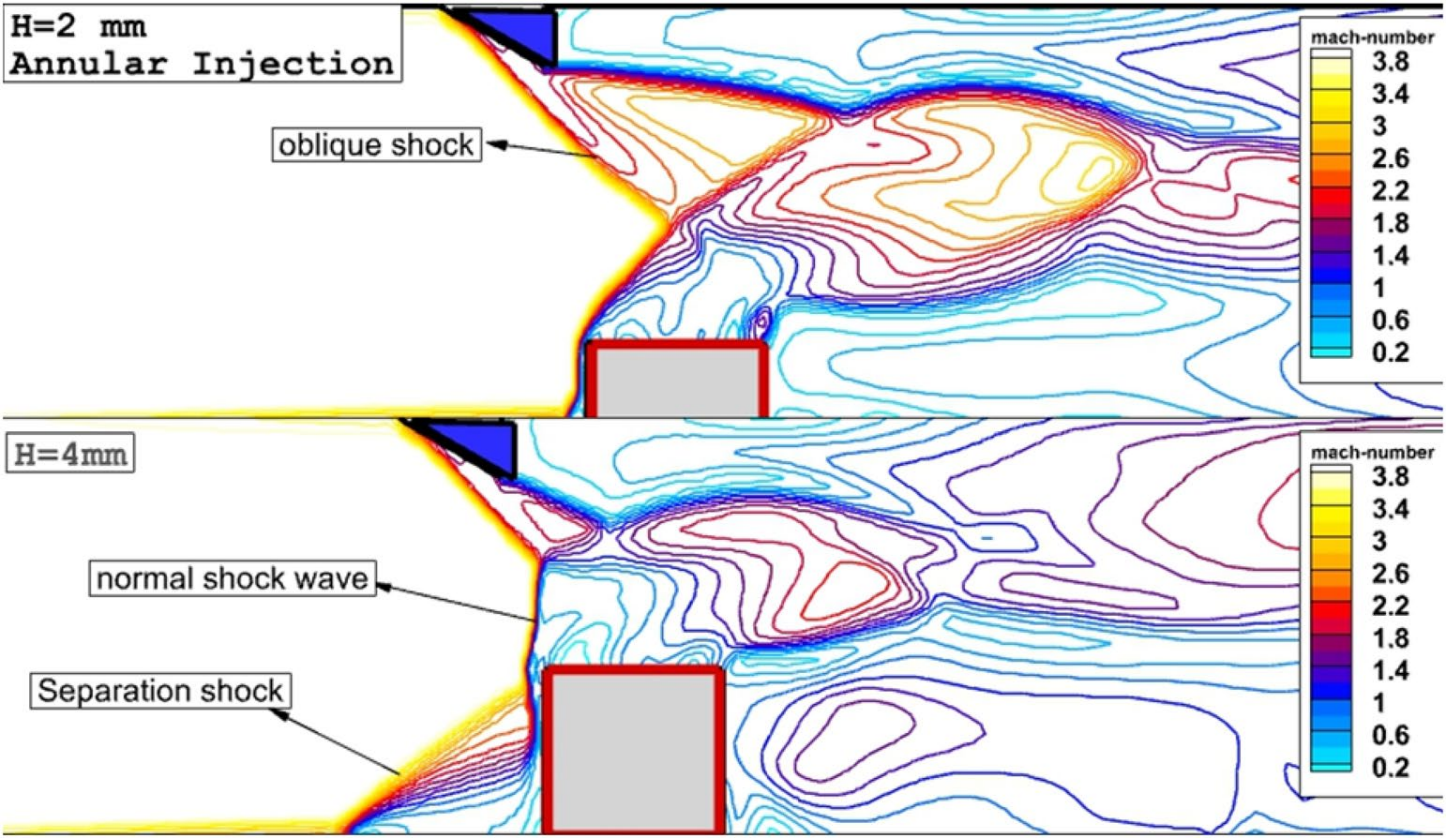
Comparison of the produced shock waves in the jet plane.

The contacts of the supersonic air stream inside the combustor with extruded nozzle and shock generator are noticed via Mach contour (Fig. 3) on the jet plane where the interactions of the jet with the air stream are high. The Mach contour shows that the altitude of the erected 4-lobe injector has a great impact on the formation of the normal shock which is noticed in higher nozzle altitudes. In the lower altitude of an extruded nozzle (H = 2 mm), oblique shock generated by the wedge shock producer interacts with bow shock originating from the upper edge of the erected nozzle at the top of the extruded injector. However, increasing the altitude of the erected nozzle induces separation shock and consequently, produces oblique and bow shock contact upstream of the injector. Hence, a normal shock wave or Mach disk is noticed in this area. The produced separation shock also creates circulation flow which has a momentous influence on the fuel mixing as it will be discussed in the next sections.

**Figure 4:**
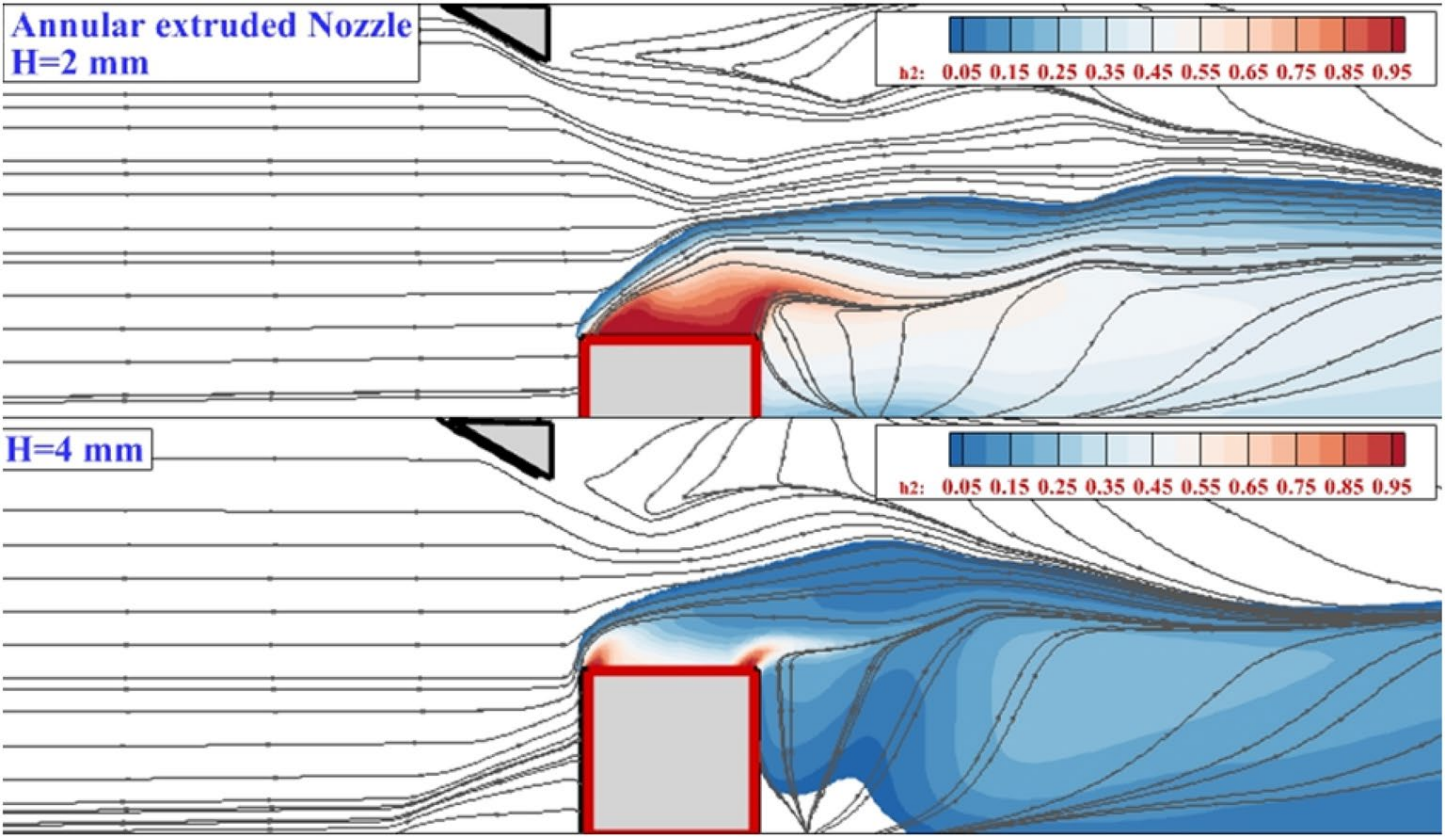
Mixing zone and flow stream in annular 4-lobe extruded nozzle.

Figure 4 demonstrates the mixing zone and flow stream of the single annular extruded nozzle for two altitudes of injector on the jet plane. As mentioned in the shock contour, the effects of the shock generator on the deflection of the injected hydrogen jet are momentous. In the low altitude (H = 2 mm), the concentration of the fuel jet is almost near the injector and the role of the vortex on the fuel dispersal is not high. As the nozzle altitude is elongated, the height of the fuel mixing is concededly improved while the fuel concentration near the nozzle is decreased. Meanwhile, the role of the vortex behind the extruded nozzle is higher.

Shock-shock interaction in supersonic flow occurs when two shock waves intersect and affect each other's properties, such as strength, angle, and position. This interaction can lead to complex changes in flow properties, including changes in pressure, temperature, and flow direction. Understanding shock-shock interactions is crucial in the design and analysis of supersonic aerodynamics, as it directly impacts the behavior and performance of vehicles and airfoil shapes in supersonic flow conditions.

**Figure 5:**
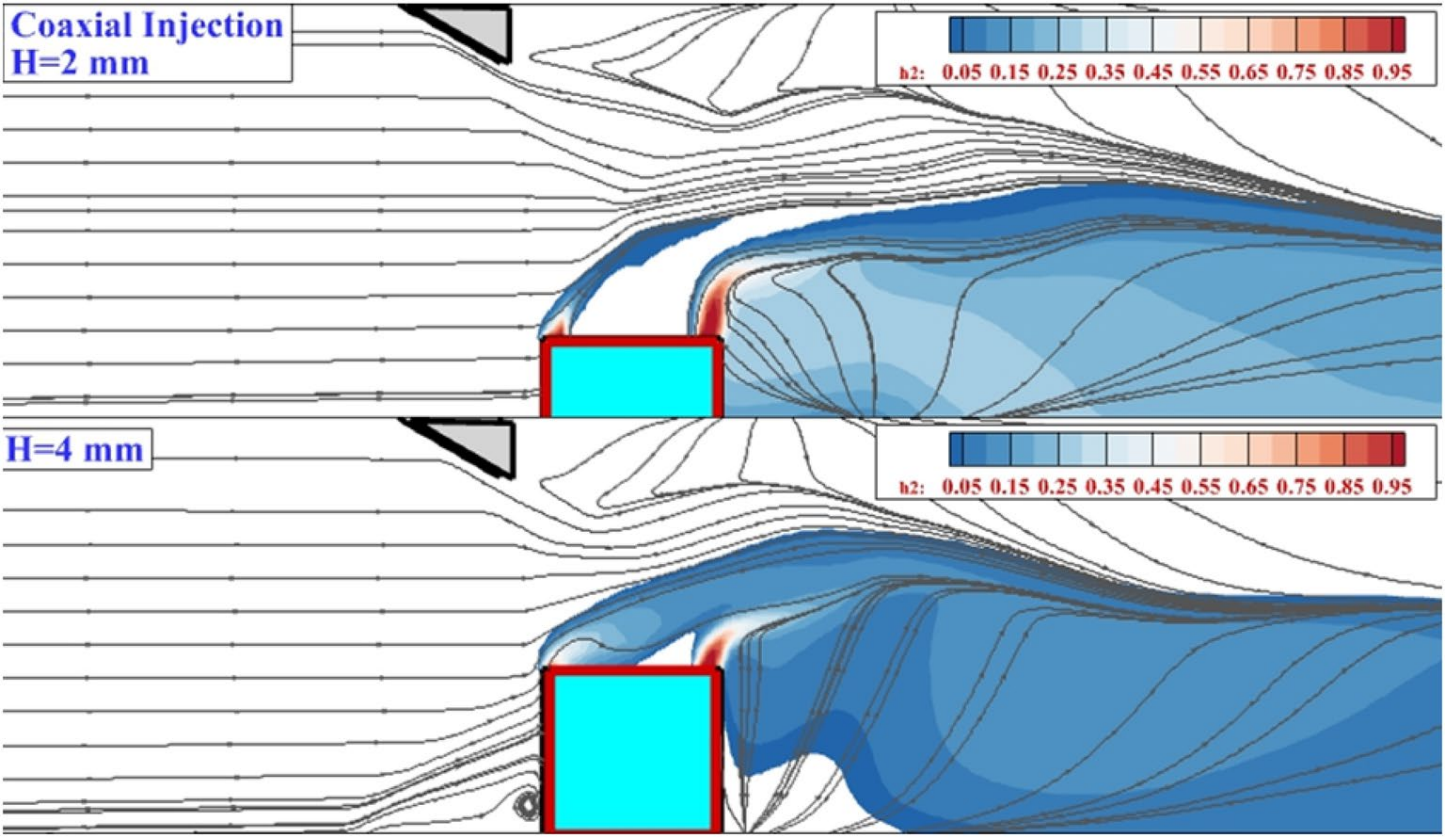
Mixing zone and flow stream in coaxial 4-lobe extruded nozzle.

The addition of the interior air jet has clear impression on interactions of the fuel penetration inside the combustor. Figure 5 demonstrates the mixing zone of the two altitudes of the erected nozzle with an internal air jet. In low altitudes (H = 2 mm), the interior air jet increases the height of the mixing region and this change is attained by the momentum of the airflow. Besides, the hydrogen mass concentration is lower than a model without interior airflow on the jet plane. Indeed, the induced oblique and bow shocks reduce the velocity of the supersonic air stream, and the flow speed behind the nozzle is reduced. Thus, the fuel jet has enough time to mix with the air stream.

**Figure 6:**
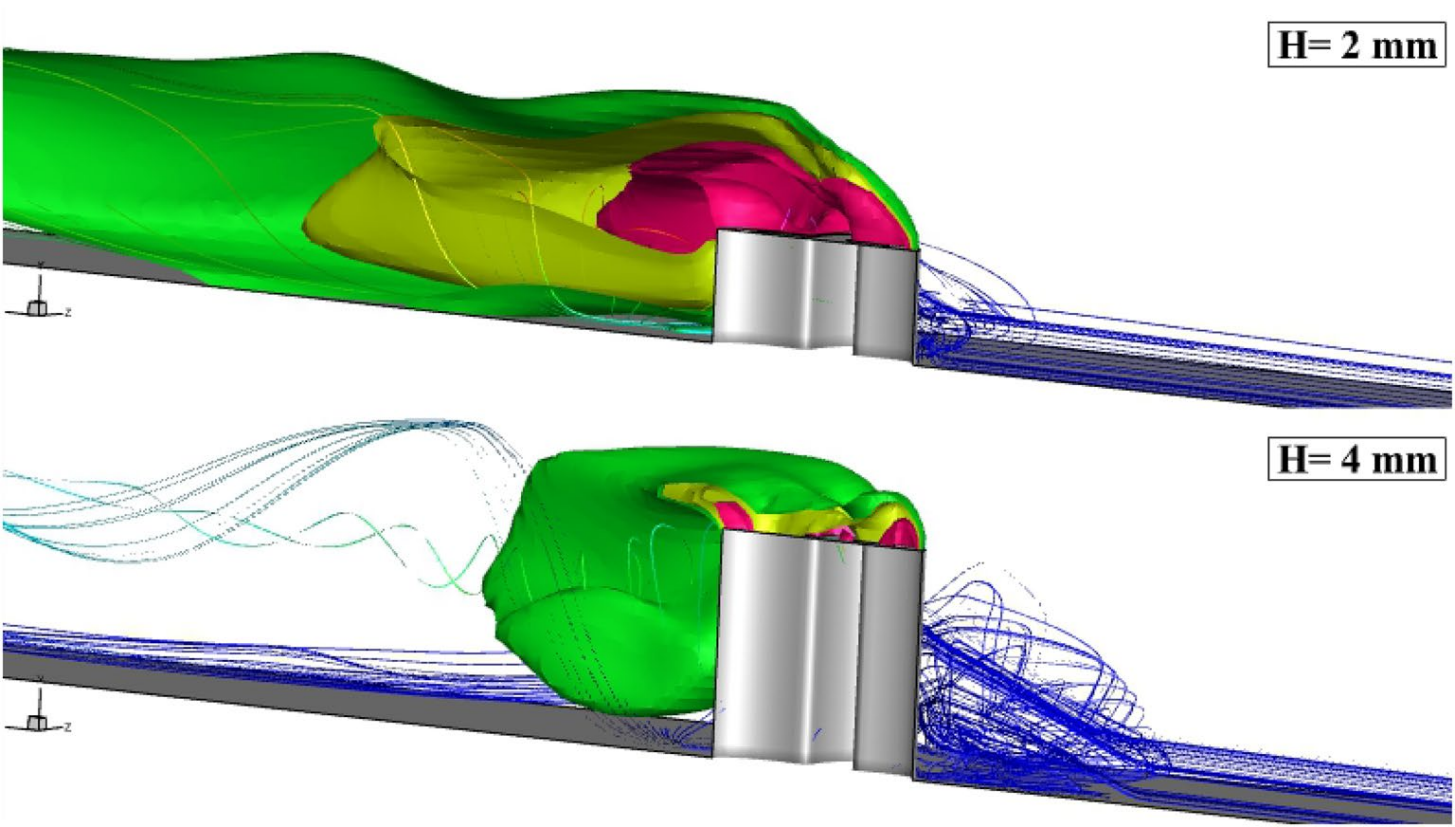
flow structure of the annular extruded 4-lobe injector (fuel concentration and flow stream).

**Figure 7:**
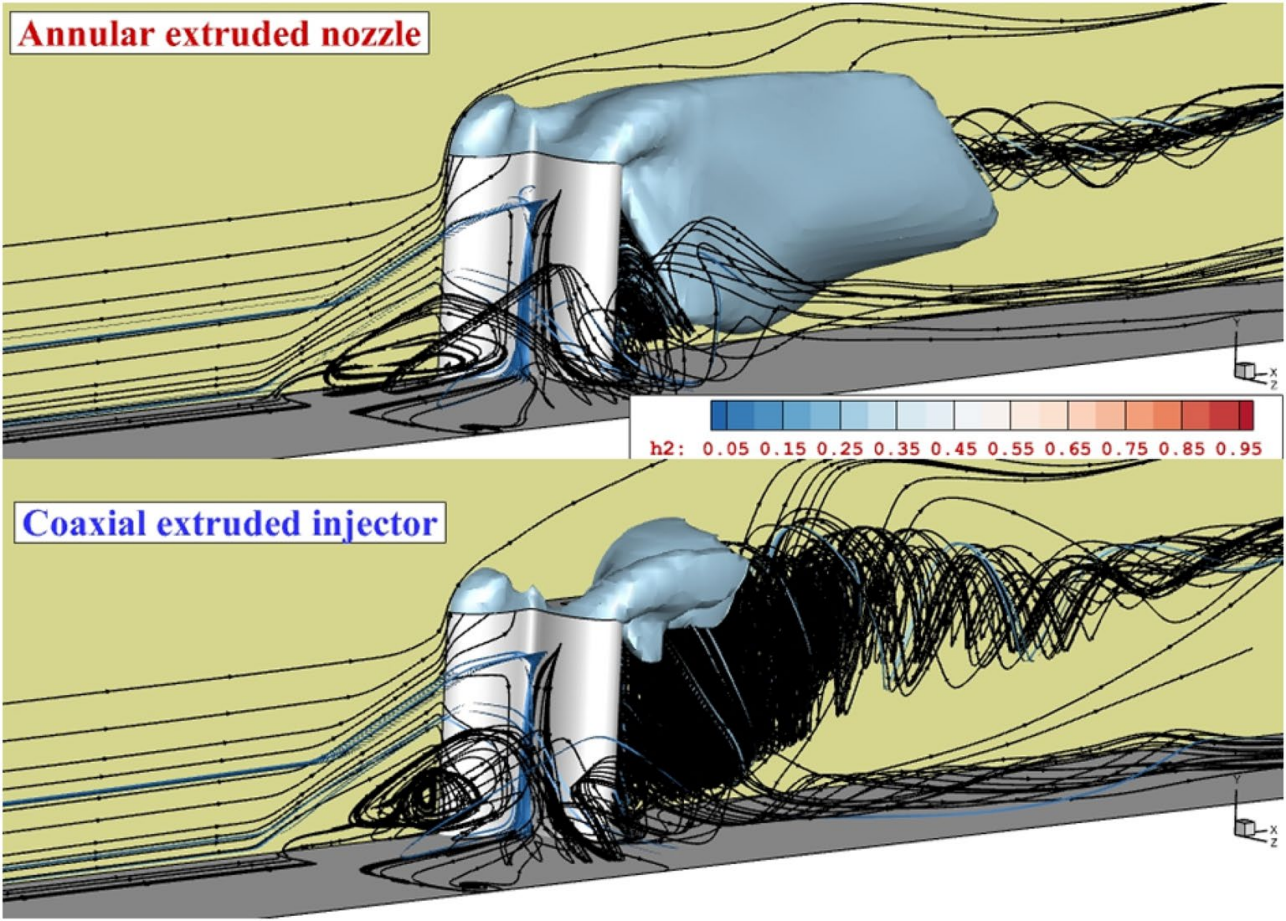
Comparison of the flow structure of the annular and coaxial extruded 4-lobe injector.

The 3-dimensional shape of the annular fuel jet injected at two different altitudes is demonstrated in Fig. 6. The supersonic air stream interactions with the erected 4-lobe nozzle result in the production of the circulation and this expanded by the elongation of the nozzle. The shape of the fuel layer reveals the structure of the released hydrogen jet under the impacts of the induced shocks upstream of the fuel nozzle. The extension of the circulated flow front and behind the nozzle verifies the role of this feature in the distribution of the hydrogen jet. The contrast of the annular and coaxial injection system on the flow and fuel jet contacts is done for the model with erected 4-lobe nozzle with H = 4 mm in Fig. 7. Due to the shape of the 4-lobe injector, the production of the upstream circulation is facilitated since the nozzle has a higher extension along the width of the domain. When an internal air jet is applied, the velocity behind the jet is decreased. Then, the trail of the upstream vortex expands freely behind the nozzle. As highlighted in the coaxial case, the flow circulation is a noticeable downside of the erected 4-lobe nozzle with an internal air jet.

**Figure 8:**
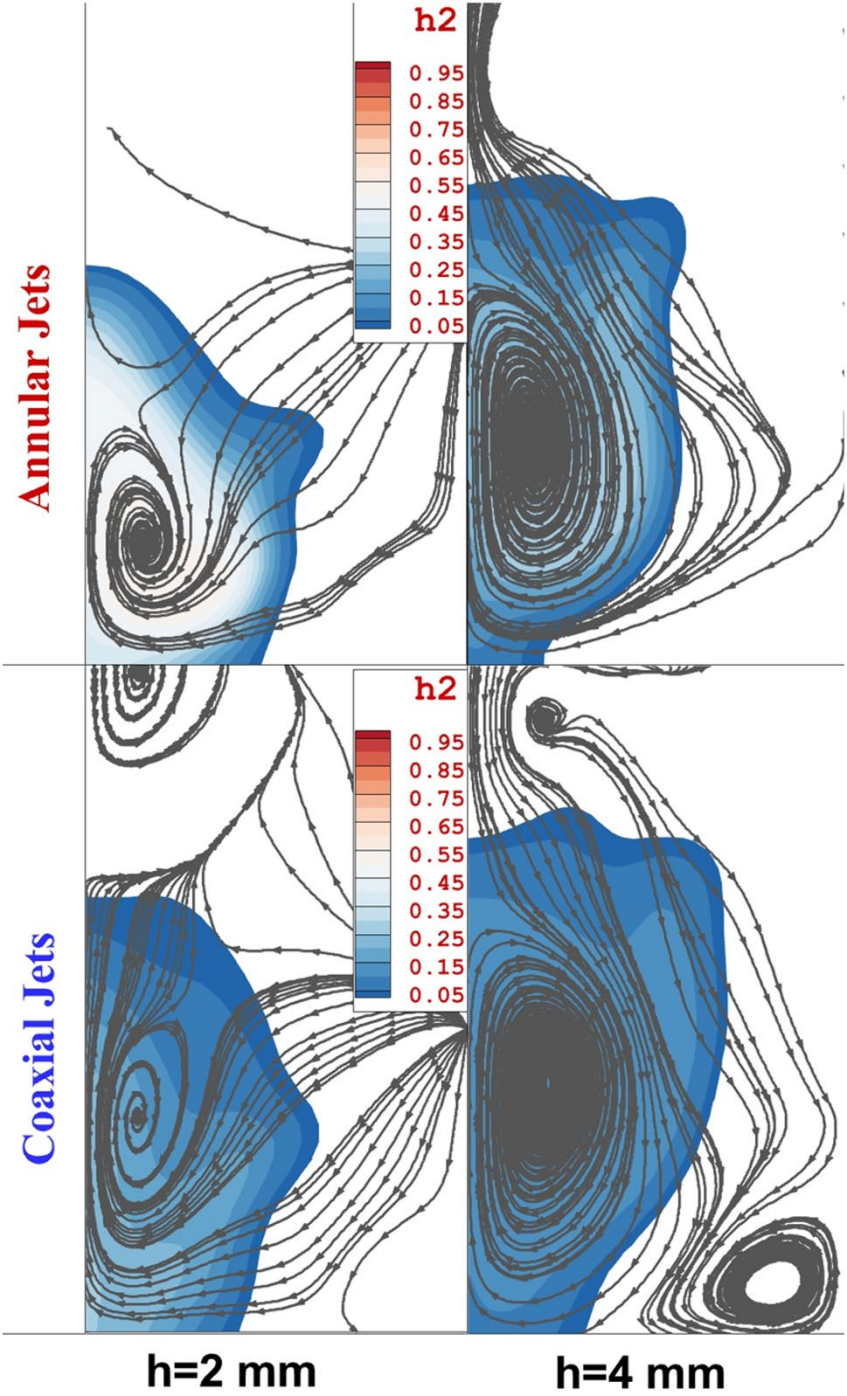
mixing zone and stream feature behind the jet.

Figure 8 displays the mixing zone and stream on the cross-section plane located 20 mm behind the 4-lobe extruded nozzle with/without an inner air jet. In the annular cases, the extension of the counter-rotating vortex in the core of the fuel jet is extended as the nozzle altitude is increased from 2 to 4 mm. Contrast of the model with the internal air jet confirms the role of the air stream on the extension of vortex pair and horseshoe vortex trailed from the upstream circulation. The latter limited the mixing zone as the altitude of the coaxial configuration is high. Horseshoe vortex is a flow pattern that occurs around a bluff body, such as a fuel injector, where the flow separates, creating a distinctive horseshoe-shaped vortex. This vortex can significantly affect fuel distribution in combustion systems. When a fuel injector is placed in a flow field, the horseshoe vortex can disturb the incoming airflow, leading to uneven fuel distribution as demonstrated in Fig. 8. This occurs as the vortex can cause localized areas of high and low fuel concentration, impacting combustion efficiency. Counter-rotating vortices are formed when two vortices with opposite rotational directions interact. In the context of fuel distribution, this phenomenon can occur in intake systems or combustion chambers. These vortices can affect fuel distribution by creating regions of turbulence and recirculation. Consequently, fuel may not uniformly mix with air, leading to uneven distribution and potentially incomplete combustion. Both the horseshoe vortex and counter-rotating vortices can disrupt the intended flow patterns within a combustion chamber or intake system. This disruption can lead to non-uniform mixing of fuel and air, potentially causing areas of fuel-rich and fuel-lean mixtures, impacting combustion efficiency, emissions, and overall engine performance.

**Figure 9:**
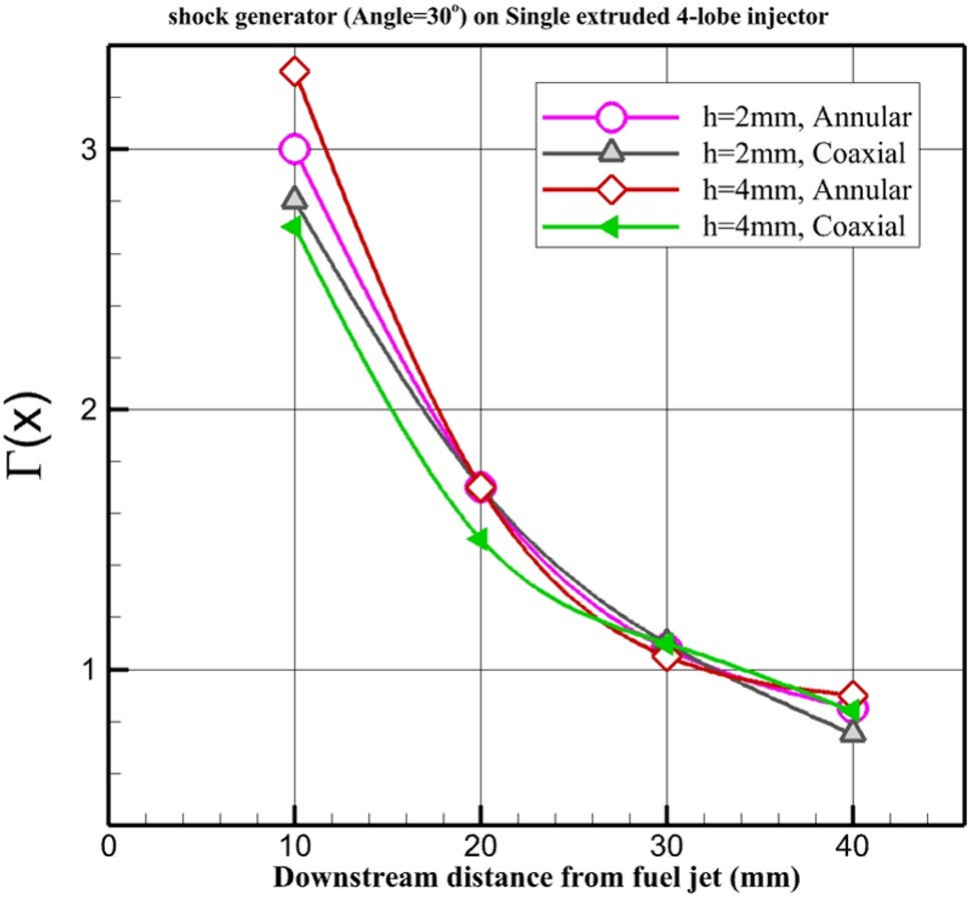
Strength of the circulation.

Figure 9 displays the circulation strength on the 4-lobe extruded model in different air and fuel configurations. The main difference in circulation power is observed near the nozzle and annular configuration with H = 4 mm having the most circulation strength among these introduced arrangements. In the second place, the annular nozzle with h = 2 mm has higher circulation strength. In the far downstream, there is no meaningful difference in circulation power. Since the difference between these models is not high, evaluation of the mixing efficiency is required to find the role of these models on the fuel distribution. The formulation for calculation of the circulation^53–55^ is as follows:

1 $$\Gamma (x)=\frac{1}{{d}_{j}^{*}{u}_{i}}\iint \left|\frac{\partial v}{\partial z}-\frac{\partial w}{\partial y}\right|{\text{d}}A$$

To calculate this factor, four planes behind the injector are selected and this formula is calculated on these plane.

The "strength of recirculation" in the context of a cross jet at a supersonic combustion chamber refers to the magnitude or intensity of the flow recirculation phenomenon that occurs due to the interaction of two or more turbulent jets. It represents the extent to which the flow is redirected or reversed in certain regions within the combustion chamber.

Understanding the strength of recirculation is crucial as it directly influences the mixing of fuel and oxidizer, combustion efficiency, flame stability, and overall combustion performance. A strong recirculation promotes better mixing, leading to enhanced combustion and heat release. On the other hand, weak or insufficient recirculation can result in incomplete combustion, flame instability, and reduced performance.

By analyzing and measuring the strength of recirculation in a cross jet at a supersonic combustion chamber, researchers and engineers can gain insights into the flow dynamics, optimize combustion chamber designs, and develop strategies to improve combustion efficiency and stability.

**Figure 10:**
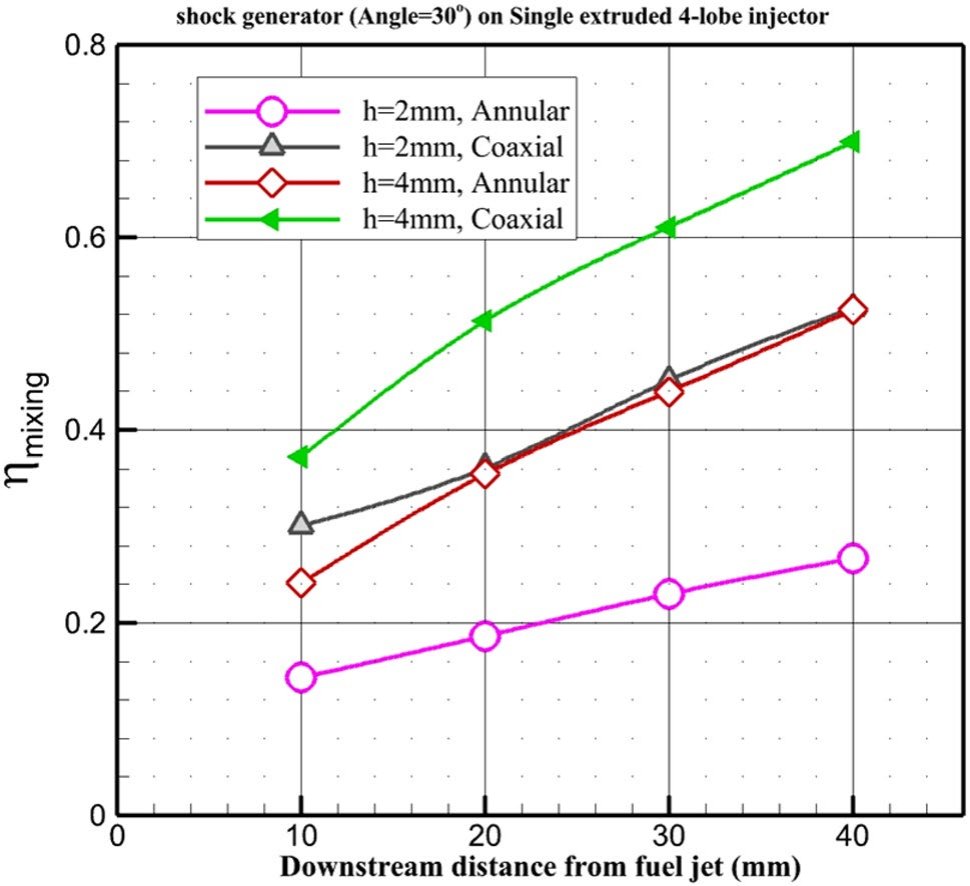
Fuel mixing behind the different injection model.

Figure 10 plots the change of the fuel mixing of the proposed arrangements for injection of the fuel from the single extruded four-lobe nozzle. Lee^53^ offered the definition for the calculation of mixing as follows:

2 $$\eta_{mix} = \frac{{\iint {Y_{{H_{2} }}^{r} }\rho u.dy.dz}}{{\iint {Y_{{H_{2} }}^{{}} }\rho u.dy.dz}}$$

where

3 $$Y_{{H_{2} }}^{r} = \left\{ \begin{gathered} Y_{{H_{2} }}^{{}} ,Y_{{H_{2} }}^{{}} \le Y_{{H_{2} }}^{st} \hfill \\ Y_{{H_{2} }}^{st} \left( {\frac{{1 - Y_{{H_{2} }}^{{}} }}{{1 - Y_{{H_{2} }}^{st} }}} \right),Y_{{H_{2} }}^{{}} > Y_{{H_{2} }}^{st} \hfill \\ \end{gathered} \right.$$

where $Y_{{H_{2} }}^{st}$ is the stoichiometric hydrogen concentration for a fuel/air mixture.

In fluid dynamics and related fields, mixing efficiency and mixing coefficient^56^ are both measures used to quantify how effectively different fluid streams mix together. However, they represent different aspects of the mixing process and are calculated differently. The formula you provided for mixing efficiency, ${\eta }_{mix}$, appears to be specific to a certain context, likely involving the mixing of two fluid streams. In this formula (2) and (3), stochastic fuel mixing ratio to total fuel concentration is evaluated regarding the mass flow rate. A higher mixing efficiency indicates more effective mixing.

The gas mixing coefficient, as you defined it, is the ratio of the mass of the mainstream entering the recirculation zone to the mass of the jet entering the recirculation zone. This coefficient is more concerned with the mass transport aspect of mixing rather than the detailed fluid dynamics within the mixing region. It provides a measure of how much of the mainstream fluid is entrained or mixed with the jet or secondary flow. Typically, this coefficient is used in applications such as combustion, where efficient mixing of fuel and oxidizer streams is crucial for combustion performance.

In summary, mixing efficiency focuses on the dynamics of the flow fields and how well they interact to promote mixing, whereas the mixing coefficient (gas mixing coefficient in this case) is more concerned with the mass transport aspects and quantifies the ratio of mass entrainment or mixing between different fluid streams.

The highest mixing efficiency is obtained in the coaxial 4-lobe configuration (h = 4 mm) with an inward air jet. The second efficient model is a coaxial configuration with h = 2 mm as well as an annular injector with h = 4 mm. The mixing performance of these two models is about 30% lower than the coaxial air jet with h = 4 nun. The least efficient model is an annular configuration with h = 2 mm. Replacing the annular nozzle (H = 2 mm) with a coaxial nozzle (h = 4 mm) would boost the fuel mixing up to 250%.

## Conclusion

In the present article, the usage of the single 4-lobe nozzle with/without an inner air jet is broadly investigated. The aim of this work is to present fuel jet flow in a combustion chamber when a single 4-lobe jet released from the extruded nozzle is placed inside the combustion chamber in the presence of a shock generator. A numerical technique with an SST turbulence model is developed for the simulation of supersonic airflow with a transverse fuel jet system. The influences of nozzle altitude and interior air jet for the development of fuel mixing are extensively examined. Besides, the flow near the nozzle is analyzed in diverse injector configurations. Based on our results, the usage of an extruded 4-lobe nozzle for the mixing of the fuel jet is known efficient. Also, increasing (double) the length of the extruded nozzle would increase the fuel mixing of annular and coaxial jets by about 90% and 45%, respectively.

## Data Availability

All data generated or analysed during this study are included in this published article.
